# Iron Supplementation and Neurodevelopmental Outcomes in Infancy and Early Childhood: A Review

**DOI:** 10.3390/children13091274

**Published:** 2026-09-19

**Authors:** Sergio Jose Torralbas Fitz, Victoria Jones Sanchez, Antonio Muñoz Hoyos, Daina E. Merino Pena

**Affiliations:** 1 Musculoskeletal Oncology Division, Department of Orthopaedics, University of Miami Health System, Miami, FL 33136, USA; sjt96@med.miami.edu; 2 Clinical Medicine and Public Health Program, University of Granada, 18016 Granada, Spain; elsamerinopena@gmail.com; 3 Facultad de Medicina, University of Granada, 18016 Granada, Spain; victoriajonessanchez@hotmail.com; 4 Departamento de Pediatría, University of Granada, 18012 Granada, Spain

**Keywords:** iron deficiency, iron supplementation, neurodevelopment, child development, pediatrics, review

## Abstract

**Highlights:**

**What are the main findings?**

- Early-life iron deficiency is associated with potential adverse effects on neurodevelopment, particularly during critical periods of brain development.
- Evidence on the neurodevelopmental effects of prophylactic iron supplementation in infants and young children is heterogeneous, with potential benefits in specific populations but insufficient evidence to establish a universal preventive effect.

**What are the implication of the main finding?**

- Prevention, early detection, and appropriate management of iron deficiency may contribute to protecting neurodevelopment during early childhood.
- Future randomized controlled trials should define the optimal timing, dose, and duration of iron supplementation and identify the children most likely to benefit.

**Abstract:**

**Background:** Iron deficiency (ID) is the most prevalent nutritional deficiency worldwide and remains a major public health concern, particularly during pregnancy, infancy, and early childhood. Because iron is essential for brain development, both insufficient and excessive iron exposure during critical developmental periods may have lasting effects on neurodevelopment. **Objective:** This study aims to review the available evidence on the effects of iron status and iron supplementation (IS) during early life on neurodevelopmental outcomes. **Methods:** A review was conducted in accordance with the PRISMA 2020 guidelines. Six electronic databases were searched independently by two reviewers. Studies investigating the association between iron status or IS and neurodevelopmental outcomes in infants and children were selected according to predefined eligibility. **Results:** The search identified 884 records, of which 15 studies met the inclusion criteria. The available evidence indicates that ID during pregnancy and early childhood is associated with poorer cognitive, motor, language, and behavioral outcomes, with some studies reporting effects that persist into later childhood and adulthood. IS reduced the risk of ID and iron deficiency anemia (IDA) in vulnerable populations. However, in children with adequate iron stores, excessive iron exposure was associated with less favorable neurodevelopmental outcomes in several studies. **Conclusions:** Maintaining adequate iron status during early life is essential for optimal neurodevelopment. The findings support targeted supplementation strategies in populations at risk of deficiency and highlight the importance of appropriate screening using biomarkers such as ferritin, hemoglobin, and inflammatory markers, including C-reactive protein. Additional longitudinal studies are needed to define the long-term neurodevelopmental effects of different supplementation strategies.

## 1. Introduction

This review provides with a concise overview of the key concepts underlying the relationship between iron metabolism and neurodevelopment. Specifically, we summarize the fundamental aspects of iron metabolism, its physiological functions, particularly within the central nervous system, and the current evidence regarding IS during early life and its implications for neurodevelopment and long-term neurological health.

Iron is an essential trace element required for most of life and plays a central role in numerous biological processes [1]. Iron metabolism encompasses the tightly regulated molecular and cellular mechanisms that maintain systemic and intracellular iron homeostasis [2]. This regulation is particularly important because iron exhibits two fundamental biological characteristics. First, iron is indispensable for a wide range of physiological functions, including oxygen transport, DNA synthesis and replication, mitochondrial energy production, cellular respiration, and intracellular signaling pathways [1]. These functions are especially critical during periods of rapid growth and brain development, when metabolic demands are high [3]. Second, despite its essentiality, iron possesses potentially toxic properties when present in excess or when its homeostasis is disrupted [2]. Through its ability to readily donate and accept electrons, free iron catalyzes the formation of reactive oxygen species via the Fenton reaction, promoting oxidative stress, lipid peroxidation, protein oxidation, and DNA damage [4]. Persistent oxidative injury can lead to mitochondrial dysfunction, ferroptosis, and cell death, highlighting the need to maintain a balance between iron availability and toxicity. Consequently, iron homeostasis reflects a central biological challenge, maintaining intracellular free iron concentrations low enough to prevent oxidative damage while ensuring sufficient iron availability to support essential physiological functions [3]. This delicate balance is achieved through molecular pathways that regulate iron absorption, transport, utilization, recycling, and storage [2].

Disruption of any component of this tightly regulated network compromises iron homeostasis and may lead to two distinct pathological states [5,6]: ID, frequently accompanied by IDA, or iron overload (IO). Both conditions have important consequences for human health. ID remains one of the leading causes of nutritional anemia worldwide, whereas IO has been associated with hereditary disorders, chronic inflammation, infectious diseases, cancer, cardiovascular disease, and neurodegenerative disorders, largely through mechanisms involving oxidative stress and tissue injury [7].

### 1.1. Iron Requirements and Epidemiological Considerations

Iron requirements vary substantially throughout life but are particularly high during periods of rapid growth, including infancy and early childhood [8]. During the first months after birth, healthy full-term infants undergo the physiological transition from neonatal polycythemia to the so-called physiological anemia of infancy. This process is accompanied by the recycling of erythrocyte iron, resulting in a marked expansion of body iron stores [8]. Consequently, total body iron content approximately doubles during the first six months of life, increasing from about 250 mg at birth to nearly 500 mg by six months of age without adverse clinical consequences [9].

After six months of age, endogenous iron stores gradually become insufficient to meet the demands of rapid growth, expanding blood volume, and ongoing neurodevelopment [8]. At this stage, infants require approximately 1 mg of absorbed iron per day. Because intestinal iron absorption averages only about 10%, the recommended dietary intake approaches 10 mg/day, a requirement comparable to that of healthy adults [8,10].

Human breast milk, although highly bioavailable, contains relatively low concentrations of iron (approximately 0.2–0.4 mg/L) [11]. Consequently, exclusively breastfed infants beyond six months of age who do not receive adequate complementary iron-rich foods or IS are at increased risk of developing ID and, ultimately, IDA [12,13].

Progressive depletion of iron stores eventually leads to absolute ID, which affects nearly 2 billion people worldwide and remains one of the most prevalent nutritional disorders globally (The World Health Report 2002: Reducing risks, promoting healthy living) [14]. According to estimates from the World Health Organization (WHO), in 2016 anemia affected 41.7% of children under five years of age, 40.1% of pregnant women, and 32.5% of non-pregnant women worldwide. The WHO further estimates that approximately 42% of childhood anemia and 50% of anemia in women are attributable to ID and are therefore potentially preventable through appropriate IS [14,15]. Nevertheless, more recent population-based meta-analyses suggest that ID may account for a smaller proportion of anemia cases, contributing to approximately 25% of childhood anemia and 37% of anemia among women [16].

Despite these differences, there is broad consensus that ID remains the most common nutritional disorder in childhood worldwide [17,18]. Beyond its hematological consequences, inadequate iron availability during critical periods of development has profound effects on brain maturation and has been consistently associated with impairments in cognitive performance, language acquisition, motor development, behavioral regulation, learning capacity, and social functioning [3,19].

### 1.2. Physiological Functions of Iron in the Central Nervous System

Iron is indispensable for normal brain development and function throughout life. Entry of iron into the central nervous system is tightly regulated at the blood–brain barrier [20,21,22]. Iron plays a pivotal role in the synthesis, metabolism, and function of several neurotransmitter systems [23]. Iron is also required for oligodendrocyte maturation and myelin formation [24]. Beyond its role in oxygen transport and erythropoiesis, iron is a fundamental regulator of numerous metabolic processes required for normal brain development and function [25]. As an essential cofactor for multiple enzymes [24], iron participates in mitochondrial electron transport and ATP production [25], neurotransmitter synthesis and metabolism [24], myelin formation [23], dendritic growth and arborization, neuronal differentiation, axonal transport, oxygen utilization, and the maturation of microglia and astrocytes [26,27,28]. Iron also contributes to synaptic plasticity by modulating calcium-dependent signaling pathways that underlie learning, memory, and neuronal adaptation [19,20].

Brain iron metabolism is dynamically interconnected with systemic iron homeostasis while remaining tightly regulated by specialized transport and storage mechanisms. Despite fluctuations in dietary iron intake and peripheral iron stores, cerebral iron concentrations are maintained within a narrow physiological range by mechanisms that regulate iron uptake, intracellular trafficking, storage, recycling, and export. This strict regulation reflects the dual requirement of providing sufficient iron to sustain the high metabolic demands of neural tissue while preventing iron-mediated oxidative injury [29,30,31].

**Figure 1:**
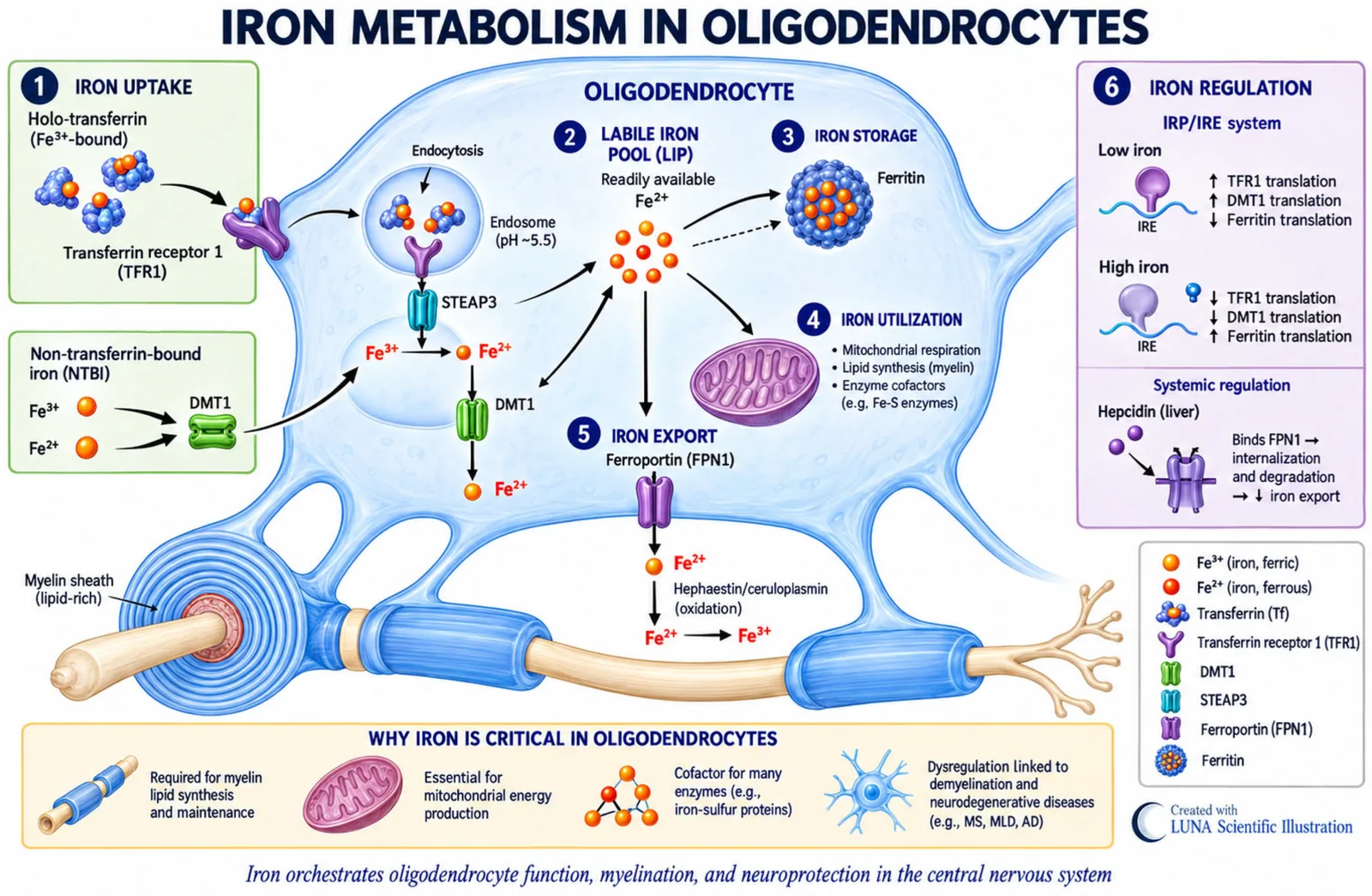
Oligodendrocyte iron metabolism. Oligodendrocytes require iron to sustain oxidative metabolism and myelin synthesis. During early development, disturbances in iron homeostasis may compromise myelination and alter the maturation of neural circuits involved in motor and cognitive function. Image created using AI.

Accumulating evidence suggests that the developmental period surrounding weaning represents a critical window during which brain iron homeostasis is established [25,28]. Iron availability during this stage appears to influence the long-term regulation of cerebral iron metabolism, with potential consequences extending into adulthood [28]. Disruption of iron homeostasis during these sensitive developmental periods may permanently alter neuronal maturation, myelination, synaptic connectivity, and cognitive function [24], thereby increasing susceptibility to neurological and neuropsychiatric disorders later in life [32,33,34]. (Figure 1).

### 1.3. Iron Depletion, Iron Deficiency Without Anemia, and Ferroptosis

Iron depletion represents the earliest stage of iron deficiency and is characterized by a reduction in body iron stores without impairment of physiological function. During this stage, serum ferritin concentrations are decreased, whereas hemoglobin levels remain within the normal range, indicating preserved erythropoiesis [35].

Progression to ID is marked by further depletion of iron stores, typically reflected by serum ferritin concentrations below 12 μg/L while hemoglobin concentrations remain ≥11 g/dL [35,36]. Although anemia has not yet developed, evidence suggests that ID at this stage may already affect infant growth, brain development, and neurocognitive function. Because of its high worldwide prevalence, particularly during infancy and early childhood, ID has become an important target for preventive public health strategies [35].

Consequently, several countries and professional organizations recommend preventive IS in selected pediatric populations, particularly those at high risk of ID [37,38,39]. Nevertheless, universal supplementation without prior screening remains controversial. Excessive iron administration in iron-replete children may interfere with the absorption of other micronutrients, alter metabolic homeostasis, affect the developing gut microbiome, and increase oxidative stress [40]. These concerns have led to growing interest in the effects of IO during early development, including its possible relationship with ferroptosis [41,42]. Ferroptosis has been implicated in several neurodegenerative disorders, including Alzheimer’s disease and Parkinson’s disease, where excessive lipid peroxidation and neuronal iron accumulation have been consistently observed in affected brain regions [43,44].

### 1.4. Neurodevelopmental Consequences of Iron Deficiency (ID)

ID during fetal life and early childhood has been associated with adverse neurodevelopmental outcomes, many of which may persist long after iron status has normalized [41]. The magnitude of these effects depends not only on the severity of ID but also on the developmental stage during which the deficiency occurs, reflecting the existence of critical windows of brain maturation [1].

ID also appears to affect memory development from the earliest stages of life [45]. Geng et al. [46] reported impaired recognition memory in two-month-old infants who presented with ID without anemia at birth. These observations are consistent with experimental evidence demonstrating that the hippocampus, one of the brain regions most critical for learning and memory [46], undergoes rapid development during the perinatal period and is particularly vulnerable to nutritional deficiencies [46,47,48].

Children with ID tend to show lower developmental quotients on standardized assessments such as the Bayley and Denver scales, poorer gross motor performance, delayed language acquisition, and deficits in expressive and receptive language. In many cases, these developmental delays persist into school age despite correction of anemia, suggesting that early ID may permanently alter neurodevelopmental trajectories [48,49,50].

**Figure 2:**
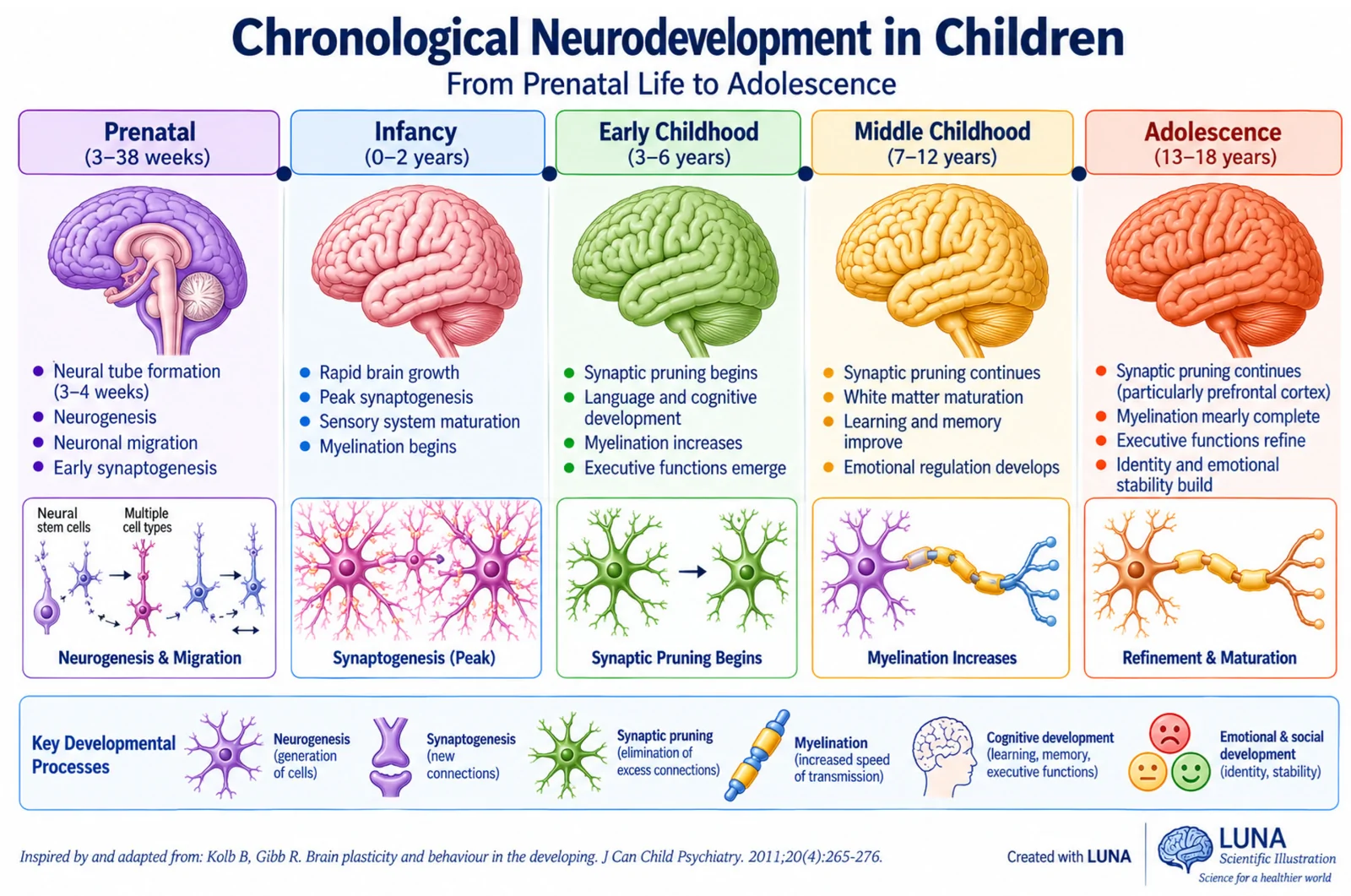
Fundamental aspects of the changes that occur in the brain during each of the key stages of neurodevelopment (Prenatal, Infancy, Early Childhood, Middle Childhood, and Adolescence). Cytomorphological changes (neurogenesis, migration, myelination, synaptogenesis, and maturation) are related to the acquisition of sensory and linguistic functions, learning, memory, executive functions, emotional maturation, and personal identity. Image created using AI [54].

Several mechanisms may contribute to the behavioral alterations observed in children with ID. On the one hand, ID may disrupt neuronal maturation [20,23,26], neurotransmitter synthesis, and myelination thereby impairing learning and cognitive processing [39,41]. In addition, affected infants often show reduced exploratory behavior, diminished environmental interaction [50], and decreased social engagement [51], which may further compromise cognitive, motor, and behavioral development through reduced environmental stimulation [52,53]. (Figure 2).

Brain regions undergoing rapid maturation during infancy appear to be especially vulnerable to inadequate iron availability [8,55]. The cerebellum is involved in motor coordination, balance, and motor learning, whereas the caudate nucleus contributes to postural control, voluntary movement, procedural learning, and the integration of cognitive and motor functions. Because these structures undergo rapid development during the first year of life, this period may represent a window of increased vulnerability in which ID can lead to persistent alterations in neural circuitry and function [55,56,57].

### 1.5. Consequences of Iron Overload (IO)

Although preventing ID remains a major public health priority (The World Health Report 2002: Reducing risks, promoting healthy living) [58,59], excessive iron accumulation may also have adverse biological consequences [60]. IO promotes oxidative stress through iron-catalyzed generation of reactive oxygen species, contributing to lipid peroxidation, mitochondrial dysfunction, chronic inflammation, and ferroptotic cell death [60,61,62]. These mechanisms have been implicated in numerous pathological conditions, including hereditary IO disorders, chronic inflammatory diseases, cardiovascular disease, cancer, and neurodegenerative disorders [63,64,65,66,67].

### 1.6. Rationale

ID remains one of the leading causes of nutritional anemia worldwide, and the World Health Organization (WHO) has consistently emphasized the importance of preventing ID, particularly among infants, young children, pregnant women, and women of reproductive age [68]. These recommendations have contributed to improvements in maternal and child health, especially in low-resource settings where ID is highly prevalent [69].

However, in regions with lower prevalence of ID, preventive IS has often been implemented broadly, sometimes without prior assessment of individual iron status [69]. Recent findings in iron biology have called this universal approach into question by demonstrating that both ID and iron excess may adversely affect neurodevelopment through distinct biological mechanisms [60].

The growing understanding of iron homeostasis, together with recent discoveries regarding ferroptosis and iron-mediated oxidative injury, suggests that IS should be guided not only by the prevention of anemia but also by the maintenance of optimal physiological iron balance. These observations suggest that the potential benefits and risks of routine IS during infancy should be reassessed [70].

### 1.7. Problem Statement

Research in molecular biology, genetics, and developmental neuroscience has improved our understanding of iron metabolism and its role in brain development [41]. In particular, growing evidence implicating ferroptosis in neurological disease has prompted reconsideration of traditional pediatric practices, including universal prophylactic IS in infants without prior assessment of iron status [60,61,65,71].

Taken together, these findings suggest that current preventive strategies should be reconsidered current preventive strategies considering contemporary knowledge regarding iron homeostasis, developmental neurobiology, and the potential consequences of excessive iron exposure during critical periods of brain maturation [60,71].

### 1.8. Aim of This Review

This review examined the available evidence on IS during infancy and early childhood for the prevention of IDA, with particular emphasis on its potential effects on neurodevelopment. This review examined whether the available evidence supports universal IS or favors more individualized strategies based on iron status and biological risk.

### 1.9. Objectives

The objectives of this review were: (1) to evaluate the extent to which prophylactic IS has been implemented in infants and young children; (2) to determine whether prophylactic IS strategies were preceded by assessment of individual iron status; (3) to critically review current evidence regarding the physiological role of iron in early brain development and neurodevelopment; and (4) to evaluate the potential effects of both ID and IO on neurodevelopment during childhood and long-term neurological health.

## 2. Methods

This review was conducted in accordance with the Preferred Reporting Items for Systematic Review and Meta-Analyses (PRISMA) guidelines 2020. The search strategy was developed following established methodological recommendations for Systematic Review [72,73]. It was registered at the University of Granada as an undergraduate thesis, not in the international registry for systematic reviews, PROSPERO.

### 2.1. Search Strategy

A comprehensive literature search was performed in the following electronic databases: PubMed, Scopus, Web of Science, SciELO, Cochrane Database of Systematic Review, and the Cochrane Central Register of Controlled Trials (CENTRAL). The primary search focused on studies published within the previous ten years. Older publications were considered when they provided relevant evidence that remained applicable.

The research question was formulated according to the PICO framework. The study population comprised infants and young children during the first years of life. The intervention consisted of prophylactic oral IS. Comparators included different supplementation regimens, doses, or standard care. The primary outcomes were the prevention of ID and IDA and their potential effects on neurodevelopment.

The search included keywords related to iron metabolism, neurodevelopment, and prophylaxis together with the corresponding Medical Subject Headings (MeSH) and Health Sciences Descriptors (DeCS). The main search terms included iron, iron metabolism, IDA, neurodevelopment, and prophylaxis. These terms were combined using the Boolean operators AND and OR, and the search syntax was adapted to the indexing system of each database.

### 2.2. Eligibility Criteria

Studies were eligible for inclusion if they fulfilled the predefined Population, Intervention/Exposure, Comparator, and Outcome (PICO) criteria. The review focused on infants and young children during the first years of life, including preschool-aged children, in whom iron supplementation, prophylactic iron supplementation, iron status, or iron deficiency was evaluated in relation to neurodevelopmental outcomes.

For the primary evidence synthesis, eligibility was restricted to original human studies providing primary data. The eligible study designs comprised randomized controlled trials and prospective or controlled interventional studies evaluating iron supplementation or prophylactic iron supplementation and reporting at least one relevant neurodevelopmental outcome. Observational studies were eligible only when they specifically addressed the predefined relationship between iron status or iron supplementation and neurodevelopment and provided data directly relevant to the review question.

Studies were required to report outcomes related to cognitive, psychomotor, motor, language, behavioural, neurological, or overall developmental function, assessed using validated developmental instruments, standardized tests, clinical assessments, or clearly defined developmental measures.

Studies were excluded when they did not meet the predefined population, intervention/exposure, comparator, or outcome criteria. We also excluded animal and in vitro studies, studies involving populations outside the predefined pediatric age range, studies without relevant neurodevelopmental outcomes, and publications that did not provide original human data.

The term “other studies” was not used as an eligibility category. Instead, eligible study designs were explicitly defined as randomized controlled trials, prospective or controlled interventional studies, and, when meeting the predefined criteria, relevant observational studies.

Secondary sources were not included in the primary evidence synthesis. Systematic reviews, meta-analyses, narrative reviews, clinical guidelines, expert opinions, editorials, letters, commentaries, protocols, and conference abstracts without sufficient primary data were excluded from the quantitative or qualitative synthesis of primary evidence.

These publications could be consulted only for contextual purposes, including background information, identification of potentially relevant primary studies, discussion of biological mechanisms, and comparison of the findings of the present review with previously published evidence. Their conclusions were not considered as independent primary evidence in the synthesis.

### 2.3. Study Selection

The study selection process was conducted according to the principles of the Preferred Reporting Items for Systematic Reviews and Meta-Analyses (PRISMA). All records retrieved from the six predefined databases were imported into the screening process, and duplicate records were removed before title and abstract screening. Two stages of screening were subsequently performed.

First, titles and abstracts were screened according to the predefined eligibility criteria. Records that clearly did not address the target population, iron-related intervention or exposure, or relevant neurodevelopmental outcomes were excluded at this stage.

Second, the full texts of potentially eligible publications were independently assessed against the complete eligibility criteria. At the full-text stage, studies were excluded when they failed to meet the predefined criteria for population, intervention/exposure, outcomes, study design, or availability of sufficient primary data. Reasons for exclusion were documented to ensure transparency and reproducibility.

**Figure 3:**
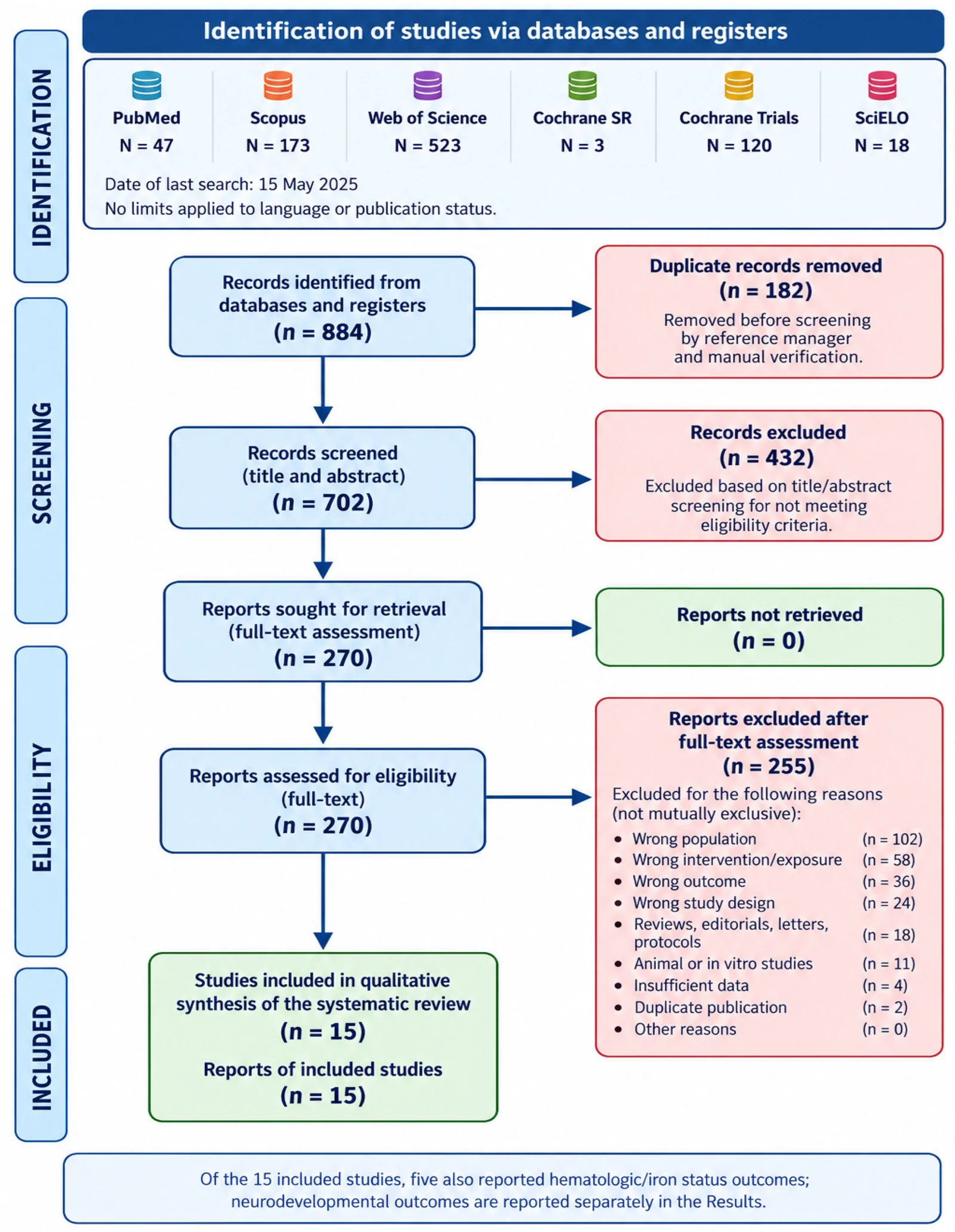
PRISMA 2020 flow diagram of the literature search and study selection process [73].

The study selection process is presented in the PRISMA 2020 flow diagram (Figure 3). The literature search identified 884 records across the selected electronic databases. After the removal of 182 duplicate records, 702 articles remained for title and abstract screening. After title and abstract screening, 432 records were excluded because they did not meet the predefined eligibility criteria, primarily due to inappropriate study populations (animal or in vitro studies, adults, or non-pediatric populations), non-original publication types, or outcomes unrelated to the objectives of this review.

The remaining 270 full-text articles were assessed for eligibility. Of these, 255 studies were excluded for one or more of the following reasons: inappropriate study design (*n* = 98), maternal-only interventions (*n* = 36), participants outside the target age range (*n* = 21), multinutrient supplementation or other ineligible interventions (*n* = 74), severe systemic comorbidities that could confound the effects of IS (*n* = 11), or failure to report relevant neurodevelopmental outcomes (*n* = 15). Ultimately, 15 prospective studies met all predefined eligibility criteria and were included in the final SR.

### 2.4. Publication Period

The electronic database searches were restricted a priori to studies published during the 10-year period specified in the review protocol. This publication-date criterion was applied consistently across all databases and during the subsequent screening process. Studies published outside this predefined period were not eligible for inclusion in the primary evidence synthesis. No discretionary inclusion of older publications based on their perceived scientific or clinical relevance was permitted. The exploration of the six databases was carried out during the period between December 2025 and April 2026.

### 2.5. Risk of Bias Assessment

The methodological quality and risk of bias of the included studies were assessed using validated tools according to study design. Randomized controlled trials were assessed using the Cochrane Risk of Bias 2 (RoB 2) tool, which evaluates bias arising from the randomization process, deviations from intended interventions, missing outcome data, outcome measurement, and selection of the reported result. For non-randomized studies, the Risk of Bias In Non-randomized Studies—of Interventions (ROBINS-I) tool was used, considering potential bias arising from confounding, participant selection, classification of interventions, deviations from intended interventions, missing data, outcome measurement, and selection of the reported result. The risk of bias assessment was conducted independently by the reviewers. Any disagreements were resolved by discussion and consensus. The results were categorized according to the recommendations of the respective instruments and were considered when interpreting the findings of the review. The Sackett/Oxford hierarchy [74,75] was used separately to classify the level of evidence associated with the included studies and was not used as a substitute for the risk of bias assessment.

## 3. Results

Table 1 summarizes the results of the literature search and study selection process based on the predefined inclusion and exclusion criteria, shows the number of records identified, duplicates removed, records screened, exclusions, and studies included in the qualitative synthesis for each database, as well as the overall data and the 15 works ultimately selected for qualitative synthesis.

Following study selection, the included articles underwent a detailed critical appraisal. Table 2 summarizes the main characteristics and findings of the included studies. Several aspects deserve attention: (1) The case studies presented in each study, which together offer a considerable sample of 7840 young children. However, the populations, settings, and interventions were highly heterogeneous, due to the very different populations, contexts, and varying interventions. (2) The type of study, where we only found 8 randomized clinical trials with different configurations, also lacks sufficient homogenization. (3) Regarding iron administration guidelines, there is no uniformity in the iron preparation used, the dosage, or the duration of administration. (4) The developmental monitoring has focused on two distinct stages: an initial stage around the first few years and a long-term stage with several assessments at ages 7, 10, 16, and 21. (5) The most relevant hematological results of the treatment. (6) The neurodevelopmental results. These aspects will be discussed below.

## 4. Discussion

This review reviewed the available evidence regarding iron supplementation during early infancy and its impact on neurodevelopmental outcomes in both the short and long term. The available evidence suggests that the effects of supplementation are strongly influenced by several factors [83], including baseline ferritin and hemoglobin levels, as well as infant-specific characteristics such as the timing of iron administration and the age at weaning [83,88,89]. The studies included in this review suggest different effects of IS according to the characteristics of the population studied: infants with increased metabolic demands—such as late-preterm infants [77], those with low birth weight, or those born to anemic mothers [76]—derive substantial benefits from IS demonstrating significant improvements in developmental quotients, behavioral outcomes, and brain connectivity [69,77,82,83,89]. In contrast, these benefits are generally not observed among healthy, non-anemic, term infants raised in stable socioeconomic environments [79,80,88]. Furthermore, when ID is identified only after the first months of life, IS administered for several months may successfully correct anemia; however, concerns remain regarding its ability to fully restore certain aspects of neurodevelopment [81,86].

These findings support the concept of the “iron paradox,” whereby both iron deficiency and iron excess may adversely affect neurodevelopment through different biological pathways [60]. Excessive exposure to high doses of iron, including prophylactic supplementation in children with normal hemoglobin concentrations, not only appears to confer no additional benefit but has also been associated with poorer cognitive performance, visuomotor integration, memory, and executive functioning, with effects that may persist into adulthood [81,84]. The apparent inconsistency among these findings suggests the existence of a U-shaped risk curve, whereby both ID and IO may adversely affect neurodevelopment due to disruptions in iron homeostasis within the developing brain [84,87]. Consequently, iron may be simultaneously essential and potentially harmful to development, suggesting that current supplementation strategies should be reconsidered collectively [78], these observations support reconsideration of universal supplementation policies and favor a more personalized and targeted approach [81], aimed at identifying periods in which supplementation may be beneficial [85], while minimizing the risks associated with excessive iron exposure [78,80,84,87].

### 4.1. Heterogeneity of Findings: High-Risk Populations Versus Healthy Infants

We will begin this section by recalling the conclusions reached in 2016 regarding iron supplementation in malaria-risk areas [90]: “Iron treatment makes little or no difference to the risk of clinical malaria (high-certainty evidence). In resource-limited settings, iron can be administered without screening for anemia or iron deficiency, provided that effective malaria prevention and treatment services are available. In settings with limited resources for malaria prevention and treatment, iron may increase the risk of clinical malaria (low-certainty evidence).”

Among all populations included in this review, late-preterm infants and infants with low birth weight consistently derived the greatest neurodevelopmental benefit from IS. In healthy late-preterm infants, supplementation with 2 mg/kg/day from the third week of life until six months of corrected age resulted in significant improvements in overall Developmental Quotient scores at one year of age, particularly in motor development and hand–eye coordination [81].

Li et al. [82] reported the use of diffusion tensor magnetic resonance imaging to demonstrate that ID in preterm infants impairs oligodendrocyte function and myelin synthesis, leading to hypomyelination of white matter [82]. ID was associated with impaired cerebellar–thalamic connectivity, which in turn predicted poorer motor performance. The integrity of this neural network was positively correlated with performance on developmental scales such as INFANIB and PDMS-2, indicating that anatomical vulnerability is associated with poorer motor outcomes [82]. This alteration appeared reversible through administration of 2 mg/kg/day of iron beginning at 40 weeks of corrected age, resulting in normalization of iron status and motor performance by six months of age among anemic infants with low ferritin levels, bringing them to levels comparable with those of non-anemic infants with adequate iron stores [82]. In infants with marginally low birth weight (2000–2500 g), early IS was not only effective in preventing anemia but also significantly reduced the risk of externalizing behavioral problems, including aggression and rule-breaking behaviors, with benefits persisting through seven years of age [77].

Similar findings have been reported in populations exposed to greater biological and socioeconomic vulnerability [12]. Among infants born to mothers with moderate anemia (hemoglobin < 10 g/dL), supplementation initiated at two weeks of age at a dose of 2 mg/kg/day independently predicted improved long-term cognitive outcomes [89]. At 24 months of age, significantly higher scores were observed in both cognitive and language domains of the Bayley-III scale compared with infants who received standard care without IS [89]. Similarly, in settings where anemia is endemic, administration of 3 mg/kg/day to children with established IDA resulted in measurable improvements within only 14 weeks, including gains in motor development, language function (vocabulary and comprehension), and manual dexterity [69]. These findings highlight the remarkable sensitivity of neurodevelopmental processes—including myelination, synaptogenesis, and hippocampal maturation—to iron status during critical periods of brain development and suggest that timely IS may mitigate damage that might otherwise become irreversible [69,89].

Furthermore, in a randomized, four-arm trial combining IS with placebo, administered to mothers and infants before and after birth [76], IS was found to have a positive effect on overall gross motor development at 9 months. The effect was similar whether supplementation was administered only during infancy or also to mothers during pregnancy [76]. The benefits were primarily related to static and locomotor skills. In addition, IS during infancy reduced the proportion of children in the lowest quartile of the locomotor subscale, regardless of whether their mothers received IS during pregnancy [76]. Several factors may explain the preferential effect of IS during infancy compared with pregnancy: Brain areas mature at different times and require iron at different rates [53]. Different motor domains (e.g., reflexes, sensory integration, postural control, motor activity, motor coordination, and planning) are supported by different brain areas and networks [76]. Complex brain areas and pathways involved in gross motor development mature more rapidly in the first year of life, thus requiring more iron and increasing their vulnerability to ID [19,20,21]. Iron is specifically necessary for oligodendrocyte function and myelin formation [20,23,46]. Consequently, neural pathways involved in the acquisition of motor skills, such as the corticospinal and corticostriatal tracts, may be more vulnerable to the effects of ID in infancy than during gestation because these pathways are not fully myelinated at birth [58]. The authors concluded that IS in infancy, with or without supplementation during pregnancy, improved scores on gross motor tests at 9 months [76].

Conversely, in high-income countries and settings with a low risk of anemia, universal IS in healthy, breastfed infants offers no added benefits [47,79]. The SIBDI clinical trial, conducted in Poland and Sweden [88], concluded that supplementation with 1 mg/kg/day does not improve psychomotor development (the primary endpoint), nor does it improve cognitive or language subscales during the first three years of life [88]. Similarly, a study conducted in a Spanish population found no significant differences in the Bailey scale for mental or psychomotor development [79]. These results suggest that additional iron intake in children from developed countries with adequate nutrition does not modify the neurodevelopmental process, even if there are improvements in biochemical status [79]. Finally, studies by the TARGet Kids group in Toronto [80] also examined children with non-anemic iron deficiency, in whom ferrous sulfate supplementation showed uncertain results in early learning compared to dietary advice [80]. On the other hand, children with chronic ID did not improve their cognitive scores after treatment, even after normalization of blood iron stores [80]. These findings suggest that ID must be prevented before it becomes chronic; once iron deficiency becomes chronic, later interventions may be less effective [50,52]. Furthermore, children with sufficient iron levels do not improve their abilities beyond their potential [85]. The lack of response to supplementation in healthy infants is largely explained by intestinal homeostasis [3,5]. Iron absorption is regulated according to body iron stores in order to limit toxicity [6]. In children without ID, this process renders additional iron therapy ineffective, since existing stores are sufficient to meet the biological requirements of vital processes such as myelination [87,88].

### 4.2. The Iron Paradox: Risks of Overexposure and the Dose–Response Relationship

Iron homeostasis remains immature during the first six months of life [8]. Infants possess underdeveloped feedback mechanisms within both the gastrointestinal tract and the central nervous system that are responsible for restricting excessive iron uptake [9]. As a result, iron may be absorbed in disproportionate amounts regardless of actual physiological requirements [91]. This limited capacity for self-regulation may lead to overexposure, disrupting the narrow homeostatic balance required during a period of rapid brain development [8,28]. Excess iron accumulation can induce neuronal oxidative stress [4], synaptic dysfunction, and cellular death, thereby impairing the maturation of neural networks within the frontal and parietal lobes [92]. Furthermore, excessive iron intake may interfere with the absorption of other essential micronutrients and alter the composition of the intestinal microbiota, contributing to additional systemic risks [86,87].

The most controversial aspect of the available evidence concerns the potentially detrimental effects of IS in children with optimal baseline iron status. Data from the Chilean cohort [84] demonstrated that healthy, non-anemic infants who received high-iron formula (12.7 mg/L) exhibited less favorable neurocognitive outcomes performance during adolescence and young adulthood, including deficits in visuomotor integration, visual memory, reading comprehension, and quantitative reasoning [84]. Moreover, a negative dose–response relationship was observed, whereby greater consumption of iron-fortified formula during infancy was associated with lower educational attainment at 21 years of age (β = −0.13, *p* = 0.048) [78,84]. The risk of overexposure appears particularly relevant among infants with high baseline hemoglobin concentrations (>125 g/L), in whom high-dose supplementation was associated with poorer arithmetic and reasoning skills, impaired response inhibition, and alterations in frontostriatal executive functioning [87].

Collectively, these findings support the concept that iron homeostasis operates within a narrow physiological range characterized by a U-shaped risk curve [3,56]. While supplementation may improve neurodevelopmental outcomes in high-risk populations with depleted iron stores [78], excess iron exposure in infants with adequate reserves may become a source of neurotoxicity because of the immaturity of key autoregulatory mechanisms [84,87].

### 4.3. Windows of Opportunity and the Developmental Cascade

The effectiveness of IS is closely linked to the temporal dynamics of brain maturation, which define both developmental periods during which the brain is especially vulnerable to iron deficiency [13,21,23]. Different brain regions mature at distinct developmental stages, and consequently, their iron requirements vary according to their specific developmental trajectories [24]. During the first months of life, essential neurodevelopmental processes take place, including myelination of the corticospinal and corticostriatal tracts [20], which are fundamental for the acquisition of motor skills [22,23]. This developmental framework helps explain the increased susceptibility of infants to ID and why supplementation administered during early infancy (between six weeks and nine months of age) results in substantial improvements in motor outcomes, particularly locomotion and postural control [53]. In contrast, maternal supplementation provided exclusively during pregnancy (from 14 weeks of gestation until delivery) does not appear to confer the same functional benefits to the child [76], suggesting that prenatal iron exposure alone is insufficient to meet the substantial postnatal demands of the developing brain [76].

Furthermore, initiating IS very early in life among biologically vulnerable infants—such as at two weeks of age—has been identified as an independent predictor of improved cognitive performance at 24 months. This likely reflects a developmental period during which the brain exhibits maximal plasticity and heightened sensitivity to iron status [89].

The concept of developmental opportunity is closely related to the developmental cascade model, which proposes that competencies acquired during early childhood provide the foundation for the emergence of more complex functions later in life [93]. ID during infancy therefore affects not only immediate developmental outcomes but also the subsequent cognitive and educational development [41,46,48,52]. Long-term follow-up data from the Chilean cohort demonstrated persistent deficits in visuomotor integration and spatial memory at 10 years of age [84]. Even relatively small impairments in these foundational skills may hinder the acquisition of more complex cognitive and academic abilities during adolescence [84]. This framework also helps explain why early-life ID may ultimately contribute to lower educational attainment in adulthood, as previously reported, through disruption of the cognitive foundations necessary to fully benefit from subsequent educational opportunities [84].

Perhaps the most clinically relevant finding emerging from the available evidence is the apparent irreversibility of some neurodevelopmental deficits following prolonged ID during early infancy [59]. Evidence suggests that when the critical window for preventing chronic IDA is missed, impairments in cognitive, behavioral, and motor functioning may persist despite subsequent correction of systemic iron stores [85]. Although ferritin and hemoglobin concentrations can normalize—and in some cases even exceed those of healthy peers—within a relatively short period, children who experienced ID during early infancy continue to demonstrate significantly poorer neurodevelopmental outcomes [53,59]. For example, deficits of six and nine points in Early Learning Composite (ELC) scores and visual perception measures, respectively, have been reported when compared with children who maintained adequate iron status throughout infancy [85].

### 4.4. Limitations of Traditional Biomarkers and Screening Strategies

Timely detection is essential for the successful implementation of preventive interventions; however, questions remain regarding the ability of current diagnostic tools to adequately identify infants at risk of ID [10]. Hemoglobin remains the most widely used biomarker for screening purposes, yet it presents important limitations [71,80]. Specifically, it lacks sensitivity for the detection of isolated iron deficiency, as hemoglobin concentrations decline only during the late symptomatic stages, once IDA has already been established and opportunities for early intervention have largely been missed [85,87]. Since iron deficiency precedes the development of systemic anemia, this delayed detection is particularly problematic because the developing brain may experience nutritional deprivation during critical periods when damage may become irreversible [71,82].

Another important consideration is the dissociation between systemic and cerebral iron status. In situations where physiological demands require prioritization of erythropoiesis over iron delivery to the brain, infants may present with normal circulating hemoglobin concentrations despite substantial cerebral ID. As discussed previously, adequate iron availability is essential for myelination and other neurodevelopmental processes [24,31,33,34]. Consequently, conventional hematological markers may not accurately reflect the amount of iron available to the developing brain and may correlate poorly with long-term neurodevelopmental outcomes [82,88].

Given these limitations, serum ferritin has been proposed as the biomarker of choice for preventive screening. Unlike hemoglobin, ferritin enables the identification of ID during its latent, preclinical stage, thereby facilitating intervention before the development of chronic deficiency and reducing the need for indiscriminate supplementation strategies [35,36,85]. Furthermore, several studies recommend the concurrent measurement of C-reactive protein (CRP) to improve diagnostic accuracy and exclude falsely elevated ferritin concentrations resulting from underlying inflammatory processes [36,80].

Table 3 and Table 4: WHO reference values for hemoglobin and ferritin in children.

Current public health strategies based solely on risk-factor assessment or routine anemia screening at 12 months of age appear insufficient [35]. The available evidence suggests moving toward universal screening approaches incorporating multiple biomarkers, including hemoglobin, ferritin, and CRP [34,35]. Such strategies have been shown to be both feasible and cost-effective, with the optimal screening window occurring between 15 and 18 months of age, when infant iron stores typically reach their physiological nadir [80,85]. In cases of non-anemic iron deficiency, clinical management should emphasize shared decision-making, balancing the potential benefits of oral IS against dietary counseling while considering the severity of deficiency and family preferences [80].

### 4.5. Public Health Implications and International Consensus

Current recommendations regarding IS during early infancy differ considerably across countries and scientific societies [37,47,50,59]. While the American Academy of Pediatrics (AAP) recommends supplementation with 1 mg/kg/day of iron for all exclusively breastfed infants beginning at four months of age, the European Society for Paediatric Gastroenterology, Hepatology and Nutrition (ESPGHAN) does not recommend routine supplementation in healthy, full-term infants with normal birth weight [10,83,88].

These discrepancies illustrate the uncertainty surrounding the optimal balance between preventing ID and avoiding excessive iron exposure [16]. The findings reviewed herein also call for reassessment of iron concentrations in infant formulas. Historically, formulas containing up to 12.7 mg/L of iron have been widely used in the United States to prevent anemia [37]. However, evidence from the Chilean cohort suggests that such concentrations may be unnecessary for infants with adequate baseline iron status [87]. Consequently, and in an effort to minimize potential long-term neurodevelopmental harm, European recommendations have progressively shifted toward substantially lower fortification levels (4–7 mg/L), prioritizing neurological safety over maximal hematological repletion [80,84].

Findings from large clinical trials conducted in both low-resource settings, such as Bangladesh [81,86], and high-income countries, including the SIBDI trial in Poland and Sweden [77], consistently indicate that universal IS provides little or no measurable benefit for healthy infants [88]. In contrast, supplementation remains a clearly neuroprotective intervention among biologically vulnerable populations [81,86]. Overall, these findings favor a transition from universal IS toward individualized, risk-based preventive strategies.

Overall, the available evidence indicates that IS cannot be considered a universally beneficial intervention during infancy [94]. Rather, its effectiveness depends on the complex interaction between baseline iron status, biological vulnerability, developmental timing, and the capacity to maintain physiological iron homeostasis [16,18,27]. These findings challenge the traditional “one-size-fits-all” approach to iron prophylaxis and support a more individualized strategy based on early identification of infants most likely to benefit from supplementation while minimizing the potential risks associated with unnecessary iron exposure [93].

## 5. Strengths and Limitations

This review has several important strengths. First, it addresses a clinically relevant and highly debated topic with direct implications for pediatric practice and public health. Second, the review was conducted in accordance with PRISMA guidelines and included a comprehensive search of multiple international databases. Third, the evidence was critically appraised according to the Oxford Centre for Evidence-Based Medicine levels of evidence, which facilitated the interpretation of the available evidence. Finally, this review integrates recent advances in iron biology—including hepcidin regulation, cerebral iron homeostasis, oligodendrocyte metabolism, and ferroptosis—with clinical evidence on neurodevelopment and relates these mechanisms to the available clinical evidence.

Nevertheless, several limitations should be acknowledged. The number of high-quality randomized controlled trials specifically evaluating prophylactic oral IS in healthy infants remains limited. Considerable heterogeneity was observed among studies with respect to study populations, iron formulations, dosages, duration of supplementation, biomarker assessment, neurodevelopmental outcomes, and follow-up periods. This heterogeneity precluded quantitative meta-analysis and limits the direct comparability of individual studies. Consequently, although the available evidence provides important clinical insights, some conclusions should be interpreted with appropriate caution until additional well-designed multicenter trials become available.

## 6. Clinical Implications

The findings of this review have important implications for pediatric clinical practice. Although ID remains a major public health concern worldwide, the available evidence indicates that prophylactic IS should not be regarded as a universal intervention. Rather, supplementation strategies should be individualized according to biological risk factors, baseline iron status, and the developmental stage of the child. An individualized approach may increase the neurodevelopmental benefits of IS while minimizing the potential risks associated with unnecessary iron exposure, thereby supporting safer and more effective preventive strategies during early childhood.

## 7. Future Directions

In terms of prevention and considering the evidence currently available, future strategies designed to reduce the incidence of IDA in childhood should incorporate screening programs based on appropriate biomarkers and, ultimately, personalized approaches tailored to the characteristics of specific populations. Such strategies are necessary to minimize potential adverse neurodevelopmental consequences arising from either iron deficiency or iron excess.

Recent findings linking alterations in iron metabolism and ferroptosis-related pathways to neurological disorders—including attention-deficit/hyperactivity disorder (ADHD), Parkinson’s disease, and Alzheimer’s disease—further emphasize the need for a deeper understanding of the role of iron in neurodevelopment and its potential long-term consequences. Particular attention should be directed toward elucidating the role of cerebral iron metabolism during the weaning period, a critical developmental stage that remains insufficiently understood.

## 8. Conclusions

ID during early life remains one of the most important preventable nutritional disorders affecting neurodevelopment worldwide. The studies included in this review indicate that IS provides clear neurodevelopmental benefits in infants at increased biological risk, including those born preterm, with low birth weight, or with documented ID. In contrast, current evidence does not support routine supplementation in healthy term infants with adequate iron stores and suggests that excessive iron exposure may have adverse long-term neurodevelopmental consequences.

These findings favor moving away from universal supplementation policies toward individualized preventive strategies based on early assessment of iron status, biological vulnerability, and appropriate biomarkers. This approach could improve neurodevelopmental outcomes while preserving the delicate balance of iron homeostasis during critical periods of brain maturation.

## Figures and Tables

**Table 1:** Results of the Literature Search and Study Selection Process, Abbreviations: SciELO, Scientific Electronic Library Online.

Database	Articles Identified	Duplicate Records Removed	Articles After Duplicate Removal	Excluded After Title Screening and Other Reasons	Studies Included in the Qualitative Synthesis
PubMed	47	11	36	31	5
Scopus	173	31	142	141	1
Web of Science	523	115	408	405	3
SciELO	18	2	16	16	0
Cochrane Systematic Reviews	3	1	2	2	0
Cochrane Trials	120	22	98	92	6
**Total**	**884**	**182**	**702**	**687**	**15**

**Table 2:** Structured summary of the studies included in the systematic review and considered in the synthesis of evidence and formulation of the final conclusions.

References	Population	Study Design	Intervention	Follow-Up	Hematological Outcomes	Neurodevelopmental Outcomes
Angulo-Barroso et al. [76]	- *n* = 1482 Infants - Children of mothers from a randomized controlled study (RCS) - Exclusion: umbilical cord ferritin < 35 µg/L - (Hebei, China)	- Randomized clinical trial with linked or sequential design in pregnancy and childhood with 4 arms: (1) Placebo/placebo, (2) Iron/placebo, (3) Placebo/iron, and (4) Iron/iron	- 1 mg/kg/day iron protein succinate solution vs. daily placebo - Duration: from 6 weeks to 9 months (approximately 7.5 months)	- At 9 months of age	- Reduction in ID and the prevalence of IDA in the direct supplementation group for infants	- Significant improvement in gross motor skills (PDMS-2) was observed exclusively among infants receiving IS during infancy (*p* < 0.001). - 36% reduction in the risk of underdeveloped motor skills. - Secondary variables (INFANIB) and (BRS-Bayley II): no significant differences, but improvement in the “head and trunk” factor in direct iron therapy.
Berglund et al. [77]	- *n* = 285 low birth weight infants (2000–2500 g) + 95 healthy infants (reference group) - Exclusion criteria: anemia at the start of the study (Hb < 90 g/L) - (Sweden)	- Randomized, double-blind clinical trial	- 0 (if placebo), 1 or 2 mg/kg/day ferrous succinate drops, dose adjusted at 12 and 19 weeks according to weight - All received dietary advice - Duration: from 6 weeks to 6 months (approximately 4.5 months)	- At 6 months (end of intervention), 3, 5 years and at 7 years of age	- Between 6 and 12 months: reduction in ID and IDA - At 7 years: no significant differences were observed between the intervention and control groups	- At age 7: No differences in IQ (WISC-IV) or executive functions (FTF) - Lower scores on external behavior problems (CBCL) in supplemented groups vs. placebo (*p* = 0.013)
Gahagan et al. [78]	- *n* = 405 adolescents - Original cohort = 835 healthy infants - Exclusion criteria: unstable family and/or social environment, anemia at 6 months (<10 g/dL Hb) or exclusive breastfeeding (<250 mL/day) - (Santiago, Chile)	- Long-term longitudinal cohort study (derived from a double-blind RCS)	- 12 mg/L (high-iron formula) vs. 2.3 mg/L (low-iron formula) - Duration: from 6 months to 12 months of age	- At 12 months (end of intervention) and in the long term at 10 and 16 years	- Childhood (12 months): The high-iron formula prevented IDA. - Adolescence (16 years): No significant evidence.	- At age 10: significantly worse scores on IQ, VMI, spatial memory, coordination, and visual perception in children supplemented with high iron. - At age 16: those supplemented with high iron scored worse on visual memory (Rey–Osterrieth Test), mathematics (WRAT-R), and reading comprehension. They also made more errors on neurocognitive and mental processing tasks. - The high-iron formula only benefited children with low initial hemoglobin levels; it worsened the development of infants who already had high initial hemoglobin levels.
Iglesias Vázquez et al. [79]	- *n* = 133 healthy infants - (Tarragona, Spain)	- Randomized, double-blind clinical trial	- Fortified formula with high doses of iron (1.2 mg/100 mL) vs. low doses of iron (0.4 mg/100 mL) - Duration: from 6 months to 12 months of age	- At 6 months and at 12 months	- Those who received the high dose of iron had a higher ferritin concentration (21.5 vs. 19.1 µg/L) and a lower prevalence of IDA (1.1% vs. 4.2%) at 12 months	- Bayley Scales (BSID-II): no significant differences were observed in MDI (99.1 vs. 95.8; *p* = 0.217) or PDI (90.8 vs. 86.6; *p* = 0.146) at 12 months
Parkin et al. [80]	- *n* = 60 children (1–3 years) with non IDA (Hb >110 g/L, ferritin < 14 µg/L) - (Toronto, Canada)	- Randomized, double-blind, placebo-controlled clinical trial	- 6 mg/kg/day liquid ferrous sulfate vs. liquid placebo; both groups received dietary advice - Duration: 4 months	- At 4 months (primary) and 12 months	- At 4 months: ferritin was significantly higher in the iron group (difference of 16.9 µg/L; *p* = 0.003), and 0% had persistent ID compared to 31% in the placebo group. - At 12 months: no significant differences between groups in ferritin or hemoglobin. No IDA	- Mullen Scales (MSEL): no significant differences in Early Learning Composite (ELC) at 4 months (difference 1.1) or 12 months (difference 4.1) - No differences in language or motor skills
Pasricha et al. [81]	- *n* = 3300 infants (7.5 and 8.5 months) - Exclusion criteria: Hb < 8 g/dL, severe acute malnutrition, known developmental delay - (Rupang, Bangladesh)	- Randomized, triple-arm, double-blind, placebo-controlled clinical trial	- 12.5 mg of elemental iron (ferrous sulfate syrup) vs. ferrous fumarate powder from MNP vs. placebo - Duration: 3 months	- At 11 months (after the intervention ended) and at 20 months (9 months post-intervention)	- At 11 months: Significant reduction in the prevalence of IDA (0.48 with syrup, 0.52 with MNP) and ID compared to placebo. - At 20 months: Benefits persisted only partially (IDA 29–34% in the iron group vs. 41% in the placebo group).	- Bailey III: no apparent effects on composite cognitive, language, behavioral or motor scores either in immediate post-intervention or at 9 months.
Li et al. [82]	- *n* = 63 premature infants (28–36 weeks of gestation) - Two groups: non-anemic with high ferritin (NA-HF); anemic with low ferritin (A-LF) - (China)	Longitudinal follow-up study with: - (MRI). - (INFANIB) at their 3 months - (PDMS) at 6 months of corrected age	- 2 mg/kg/day of oral iron - Duration: from 40 weeks of corrected age, for 6 months	- At 3 months and 6 months of corrected age	- At 3 months: Group A-LF showed significantly lower ferritin levels. - At 6 months: The initial differences in Hb and serum ferritin between the groups disappeared.	- At 3 months: the A-LF group had lower motor scores (INFANIB) and weaker cerebellar–thalamic structural connectivity. - At 6 months: there were no significant differences in PDMS-2 scores between groups.
Luciano et al. [83]	- *n* = 66 healthy late-preterm infants (34–36 weeks) - Final sample = 52 infants (lost due to treatment intolerance/loss to follow-up) - (Italy)	- Randomized, double-blind, placebo-controlled clinical trial	- 2 mg/kg/day iron pidolate supplementation vs. placebo - Duration: from 14 days of life to 6 months of post-conception age	- At 6 months and 12 months of post-conception age	- At 12 months, no child in the supplemented group presented with anemia, 2 cases of anemia in the placebo group. - Hemoglobin levels were similar in both groups.	- Griffiths Scale (GMDS-II): higher total Developmental Quotient (DQ) in iron group (121.4 vs. 113.2; *p* < 0.01) and higher scores in motor (*p* < 0.05), coordination (*p* < 0.01) and social (*p* < 0.02) subscales.
East et al. [84]	- *n* = 443 young adults (mean age 21 years) - Original cohort: 1657 healthy infants (6–12 months) - (Chile)	- Long-term longitudinal cohort study (derived from a double-blind preventive RCT)	- Iron-fortified formula (12.7 mg/L) vs. low-iron formula (2.3 mg/L) - Duration: 6 to 12 months of age	-At 10 and at 21 years old	- At 12 months, prophylactic iron supplementation resulted in the prevention of iron deficiency anemia	- At age 10 (WISC-R, VMI, KABC): the high-iron fortified formula was associated with worse neurocognitive performance: spatial memory, IQ, and motor road integration. - At age 21 (CogState, TMT, DERS): the high-iron fortified formula was associated with lower visual and verbal memory, slower visual learning (β = −0.12, *p* = 0.076), lower emotional awareness, and lower educational attainment (β = −0.13, *p* = 0.048).
Gingoyon et al. [85]	- *n* = 116 healthy children (12–40 months of age): 41 with chronic ID, 75 with iron sufficiency) - (Toronto, Canada)	- Prospective observational study (with nested randomized trial for NAID)	- Children with (IDA) received IS. Children with non-anemic iron deficiency (NAID) were randomized to receive oral iron versus placebo. - All treatment groups received ferrous sulfate at a dose of 6 mg/kg/day. - Children with normal iron levels did not receive treatment. - All participants received dietary counseling. - Duration: 4 months	- At the start of the study (prior to intervention), 4 months and 12 months post-intervention	- At 4 months, ferritin levels improved in the group with chronic deficiency (48.1 vs. 31.4 µg/L; *p* = 0.03). - No differences were observed at 12 months. - There were no significant differences in hemoglobin levels.	- A significantly lower ELC score (Mullen Scale) was observed in children with chronic deficiency: - 6.4 points at 4 months (*p* = 0.04) and - 7.4 points at 12 months (*p* = 0.03). - The most notable deficiency was in visual perception at 12 months (−8.9 points; *p* < 0.001).
Larson et al. [86]	- Subsample of 412 children (3rd month of study) and 374 (12th month) from the BRISC trial: 8 months of age (43.9% anemic, 26.7% with iron deficiency at baseline) - (Bangladesh)	- Randomized, triple-arm, double-blind, placebo-controlled clinical trial (neurocognitive substudy using EEG)	- 12.5 mg/day iron syrup (ferrous sulfate) vs. MNP vs. placebo daily, starting at 8 months - Duration: 3 months	- At 11 months (3 months post-intervention) and at 20 months (12 months post-intervention)	- Improvement in hemoglobin and serum ferritin levels was observed in the intervention groups (iron and MNP) compared to the placebo group at 3 months (*p* < 0.001). - At 12 months, improvements were maintained in the syrup group, but only ferritin remained elevated in the MNP group.	- Resting electroencephalography (EEG) - At 3 months: iron syrup produced an increase in the strength of the mu-alpha band (motor maturity), comparable to the placebo effect (*p* = 0.003). - No changes were observed in those supplemented with MNP. - However, the effect did not persist at the 12 month follow-up.
East et al. [87]	- *n* = 562 adolescents (mean age 16 years) - Initial cohort: 1010 healthy, non-anemic infants (6 months of age, Hb > 100 g/L) - (Chile)	- Long-term longitudinal cohort study (derived from a double-blind RCT)	- Iron-fortified formula (12.7 mg/L) or iron-fortified vitamin drops (15 mg/day) vs. iron-free cow’s milk or iron-free vitamins - Exclusion criteria: development of iron deficiency anemia during the study, subsequent treatment with therapeutic doses of oral iron - Duration: from 6 to 12 months of age	- At 12 months, 18 months and neurocognitive assessment at 16 years	- In childhood, the supplemented group had seven times less IDA than the group without iron (4.5% vs. 31.7%). - An increase in ferritin levels was observed in supplemented infants, even if their initial levels were above normal.	- The supplemented group performed worse on the VMI scale (visual–motor integration), *p* = 0.017; on the WISC-R scale (matrix reasoning), *p* = 0.031; and on the TMT and WSCT scales (neurocognitive tasks). - Negative interaction: Infants supplemented with initial Hb > 125 g/L performed worse on reasoning and arithmetic (WRAT-R), (β = −0.11, *p* = 0.039).
Svensson et al. [88]	- *n* = 221 healthy, full-term infants (>2500 g), 4 months of age, non-anemic, breastfed (>50%) - Final sample: 200 children at 12 months - (Poland and Sweden)	- Randomized, pragmatic, double-blind, placebo-controlled clinical trial - (SIBDI Study)	- 1 mg/kg/day of iron supplement (microencapsulated ferric pyrophosphate) vs. placebo (maltodextrin) - Duration: from 4 to 9 months of age	- At 12, 24 and 36 months	- At 12 months: there was no reduction in the risk of ID (RR 0.46) or IDA (RR 0.78)	- Bayley-III Scale: showed no significant effects on psychomotor (aMD −1.07), cognitive (aMD −1.14), or language (aMD 0.75) development at 12 months. - At 24 and 36 months, no significant benefits were found between groups.
Tiwari y Kukreja [89]	- *n* = 240 full-term infants born to mothers with moderate anemia (Hb < 10 g/dL) - (India)	- Prospective birth cohort study with random assignment	- 2 mg/kg/day of daily oral iron vs. standard care (without routine supplementation) - Dosage: from 2 weeks of age to 6 months	- At 6, 12 and 24 months	- At 6 months: the group supplemented with iron had significantly higher levels of hemoglobin (11.2 vs. 10.1 g/dL; *p* < 0.01) and ferritin (42.3 vs. 28.7 ng/mL; *p* < 0.001). - In addition, there was a lower incidence of iron deficiency anemia (12% vs. 31%) in the supplemented group.	- At 24 months: the supplemented group showed significantly higher cognitive scores on the Bayley-III scale (105.8 vs. 99.3, *p* < 0.001). - Language scores were also higher (98.5 vs. 94.6, *p* = 0.01). - The study concluded that early iron supplementation is an independent predictor of better scores at 24 months (β = 0.28, *p* = 0.002).
Mathur y Gyani [69]	- *n* = 52 infants 26 infants with iron deficiency anemia (Hb 8–10.9 g/dL) and 26 non-anemic infants (Hb > 11.0 g/dL) - Ages 6–24 months - (India)	- Prospective comparative pre-post study	- 3 mg/kg/day of ferrous ascorbate syrup in the anemic group vs. 5 mg of folic acid (placebo) in the non-anemic group - Duration: 14 months	- At the beginning and at 14 weeks	- Anemic group: anemia corrected in 84.6% and ferritin normalized in 80.7% - Non-anemic group: development of anemia in 42% and decrease in ferritin levels (from 34.7 to 13.4 µg/L)	- Assessment with DASII (Bayley adaptation) - A significant improvement was observed in the DMoQ (motor quotient) scale in the anemic group (*p* = 0.01) and in the mental clusters of language (*p* = 0.033) and manual dexterity (*p* = 0.005)

Keys to abbreviations: Wechsler Intelligence Scale for Children (WISC-IV). Child Behavior Checklist (CBCL) and Five to Fifteen (FTF). Intelligence quotient (IQ). Test of Visual–Motor Integration (VMI). The Wide Range Achievement Test-Revised (WRAT-R) is a measure of academic achievement. The Rey–Osterrieth Complex Figure Test, a commonly used neuropsychological test, measured visual perceptual ability and visual memory (ROCFT). Math computation arithmetic achievement from the (WRAT). The Bayley Scales for Infant Development–Second Edition (BSID–II). The BSID provides a mental development index (MDI) (to assess memory, habituation, problem solving, early number concepts, generalization, classification, vocalizations, and language and social skills) and a psychomotor development index (PDI). Early Learning Composite (ELC). Mullen Scales of Early Learning (MSEL). Multiple micronutrient powders (MNPs). Non-anemic with high ferritin (NA-HF). Anemic with low ferritin (A-LF). Infant Neurological International Battery (INFANIB). Peabody Developmental Motor Scales (PDMS). Griffiths Scale (GMDS-II). Developmental Quotient (DQ). Wechsler Intelligence Scale for Children Revised (WISC-R). Beery-Buktenica test of Visual–Motor Integration (VMI). Kaufman Assessment Battery for Children (KABC) spatial memory. CogState Computerized Brief Battery (CogState). Trail Making Test (TMT). Difficulties in Emotion Regulation (DERS. Electroencephalography (EEG). Mean difference (MD). Adjusted mean difference (aMD). Toddler Development (Bayley-III). Relative risk (RR). Developmental Assessment Scales for Indian Infants, which is an Indian adaptation of Bayley Scales of Infant Development (DASII). Motor Development Quotient (DMoQ) and Mental Development Quotient (DMeQ).

**Table 3:** Hemoglobin (g/L): WHO 2011. 2024 cut-offs for anemia [14].

Age Group	Hemoglobin (g/L)			
	No Anemia (g/L)	Mild (g/L)	Moderate (g/L)	Severe (g/L)
6–23 months	≥105	95–104	70–94	<70
24–59 months	≥110	100–109	70–99	<70
5–11 years	≥115	110–114	80–109	<80
12–14 years	≥120	110–119	80–109	<80

Source: World Health Organization. Guideline on haemoglobin cutoffs to define anaemia in individuals and populations. Geneva: WHO; 2011. 2024 [14].

**Table 4:** Serum ferritin (µg/L): WHO 2020 cut-offs for iron deficiency [15].

Age Group	Apparently Healthy Children (µg/L)	Children with Infection or Inflammation (µg/L)
6–23 months	<12	<30
24–59 months	<12	<30
5–11 years	<15	<70
12–14 years	<15	<70

Note. Serum ferritin is an acute-phase reactant. In the presence of infection or inflammation, the WHO recommends using a higher ferritin threshold for iron deficiency (≥30 µg/L in children <5 years) and considering inflammatory markers such as C-reactive protein (CRP) and/or α1-acid glycoprotein (AGP). Source: World Health Organization. WHO guideline on use of ferritin concentrations to assess iron status in individuals and populations. Geneva: WHO; 2020. Units: hemoglobin, g/L; serum ferritin, µg/L [15].

## Data Availability

No new data were created or analyzed in this study. Data sharing is not applicable to this article.
